# Preoperative Devascularization of Choroid Plexus Tumors: Specific Issues about Anatomy and Embolization Technique

**DOI:** 10.3390/brainsci11050540

**Published:** 2021-04-25

**Authors:** Valentina Baro, Joseph Domenico Gabrieli, Giacomo Cester, Ignazio D’Errico, Andrea Landi, Luca Denaro, Francesco Causin

**Affiliations:** 1 Academic Neurosurgery, Department of Neuroscience, University of Padova, 35100 Padova, Italy; andrea.landi@unipd.it (A.L.); luca.denaro@unipd.it (L.D.); 2 Neuroradiology Unit, University of Padova, 35100 Padova, Italy; josephdomenico.gabrieli@aopd.veneto.it (J.D.G.); giacomo.cester@aopd.veneto.it (G.C.); ignazio.derrico@aopd.veneto.it (I.D.); francesco.causin@aopd.veneto.it (F.C.)

**Keywords:** tumor embolization, pediatric neurosurgery, pediatric brain tumor

## Abstract

(1) Background: Surgical treatment of choroid plexus tumors is challenging, burdened by a notable risk of bleeding. Neoadjuvant chemotherapy and preoperative embolization have been attempted, with encouraging results; however, the consensus on these procedures is lacking. (2) Methods: We present a case of a 10-month-old girl who underwent preoperative embolization of a hemorrhagic choroid plexus carcinoma of the lateral ventricle via the anterior choroidal artery, followed by total resection. (3) Results: The endovascular procedure was successfully completed, despite the rectification of the anterior choroidal artery associated with the absence of flow proximal to the plexal point. Minimal bleeding was observed during resection and the patient remained neurologically intact. (4) Conclusions: The time from entrance to exit in the anterior choroidal artery should be monitored and regarded as a potential ‘occlusion time’ in this specific group of patients. Nevertheless, our case supports the feasibility and effectiveness of preoperative embolization of a choroid plexus carcinoma of the lateral ventricle via the anterior choroidal artery, without complications. Furthermore, we suggest the use of a fast-embolic agent, such as N-butyl cyanoacrylate glue, as the preferred agent for this specific pathology and patient population.

## 1. Introduction

Choroid plexus tumors (CPT) are rare neoplasms of neuro-ectodermal origin, accounting for less than 1% of all brain tumors and 1%–4% of all pediatric brain tumors. Children younger than 2 years of age are mostly affected, and the incidence of the choroid plexus carcinoma (CPC) is 20%–40% of CPT in patients under 18 months of age [1,2,3]. Choroid plexus papilloma (CPP) is the benign form of these tumors—total resection is curative without adjuvant chemotherapy—confirmed by a 5-year overall survival of 100% [2]. In contrast, the complete resection of CPC is often hindered by the hypervascularity of the lesion and the usual invasion of the contiguous brain parenchyma, leading to a high rate of subtotal resection and recurrences with a 5-year overall survival of 27%, despite adjuvant treatments [1,4,5]. The mortality rate is predominantly due to intraoperative significant blood loss in children with limited circulating volume, and it has been reported to be as high as 13.3% [4,6].

The use of neoadjuvant chemotherapy in CPC has been reported as an approach that leads to a decrease in blood loss in patients undergoing surgery [7]. Preoperative embolization, albeit limited to a small number of patients, has been attempted since the end of the 90s, with encouraging results in terms of reducing blood loss, which, in turn, reduces morbidity and mortality and increases the likelihood of total resection (Supplementary Table S1) [4,8,9,10,11,12,13,14,15]. However, the small and tortuous vasculature of small children, and the typical involvement of eloquent arteries, represent a significant hindrance. In this paper, we describe the case of a 10-month-old patient with a right lateral ventricle choroid plexus carcinoma who underwent successful preoperative embolization. In addition, the relevant literature is reviewed and discussed.

## 2. Case Description

**Figure 1:**
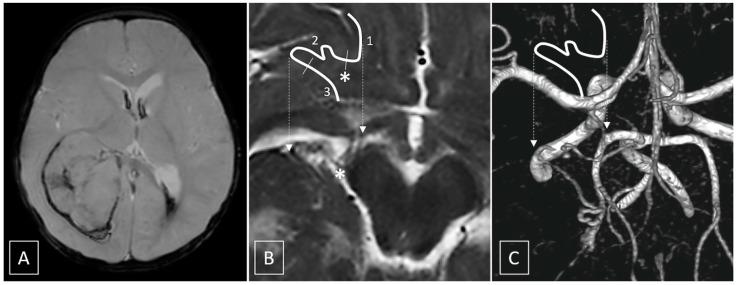
(**A**) Gradient-echo image shows intralesional, perilesional and intraventricular hemorrhagic components. (**B**,**C**) T2 axial (**B**) and Volume Rendering of ToF MRA (**C**) show the course of the anterior choroidal artery. The shifted white line represents, schematically, the course of the artery; dashed arrows indicate the exact location of the highlighted vessel. Segment 1 is the cisternal portion of the artery which courses across the carotid, crural and ambient cysterns, up to the choroidal fissure, where it enters the ventricle; the asterisk * corresponds to the plexal point that marks the entrance in the ventricle, and segment 2 is the intraventricular segment. Segment 3 is the tumoral segment of the artery running along the surface of the tumor (see Figure 4 for the complete anatomical description).

A 10-month-old female with regular neurological development and no previous medical history was admitted to our hospital for an acute onset of vomiting, followed by a progressive deterioration of her neurological status, requiring tracheal intubation. The computed tomography scan showed a large right intraventricular mass with recent bleeding associated with moderate midline shift. Magnetic resonance imaging (MRI) with contrast media and time-of-flight magnetic resonance angiography (ToF MRA) confirmed the presence of a large, hemorrhagic, intensely enhancing intraventricular mass, centered in the atrium of the right lateral ventricle, without invasion of the adjacent brain parenchyma, fed by a moderately enlarged anterior choroidal artery (Figure 1). Whole spine MRI excluded signs of cerebrospinal fluid seeding. The patient was scheduled for the following day to perform endovascular embolization and surgical excision.

**Figure 2:**
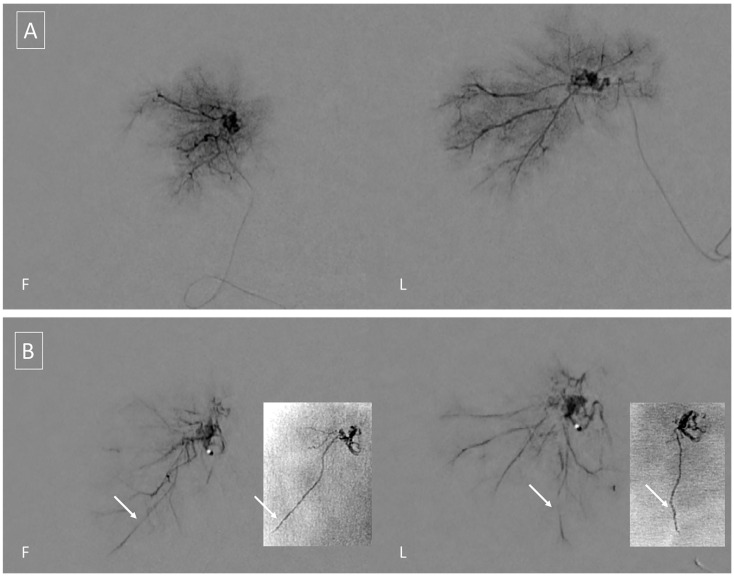
(**A**) Tumoral blush during superselective free-flow contrast injection in Frontal (F) and Lateral (L) views. (**B**) Forced injection of contrast media is performed to test for the presence of dangerous anastomosis. By comparison, with injection A, note the appearance of an additional vessel which corresponds to the course of the posterolateral choroidal artery. Unsubtracted images (small quadrants) of the final glue cast show penetration in the vascular bed of the tumor, and in the right posterolateral choroidal artery; penetration in the latter should be anticipated, even in the absence of enlarged posterior choroidal feeders, and should be carefully monitored to avoid potential complications.

A preoperative three vessel angiography (bilateral internal carotid artery and dominant vertebral artery) confirmed the presence of a slightly enlarged right anterior choroidal artery feeding the tumoral blush. No feeders were observed from the posterior circulation; embolization was obtained with n-butyl cyanoacrylate (NBCA) superselective injection (0.4 mL) via a microcatheter (Magic 1.2F-Balt, Montmorency, France; aided by a microguidewire Hybrid 0.008-Balt, Montmorency, France) navigated near the tumor pedicle, distally, to the plexal point of the anterior choroidal artery (Figure 2). The positioning of the microcatheter distally, in the anterior choroidal artery, determined, as expected, a rectification of the artery, and a panoramic injection (guiding catheter Envoy 5F-Codman Neuro, Raynham, MA-positioned in the internal carotid artery) revealed an absence of flow within the anterior choroidal artery. The total time that elapsed between micro-catheterization, glue injection and retrieval of the microcatheter was 6 min. The subsequent control angiography revealed the exclusion of the tumor blush and a re-opening of the anterior choroidal artery. Total contrast media of 30 mL (300 mgI/mL), fluoroscopy time was 11 min, total series of 9, and cumulative DAP 20 710 mGycm2.

**Figure 3:**
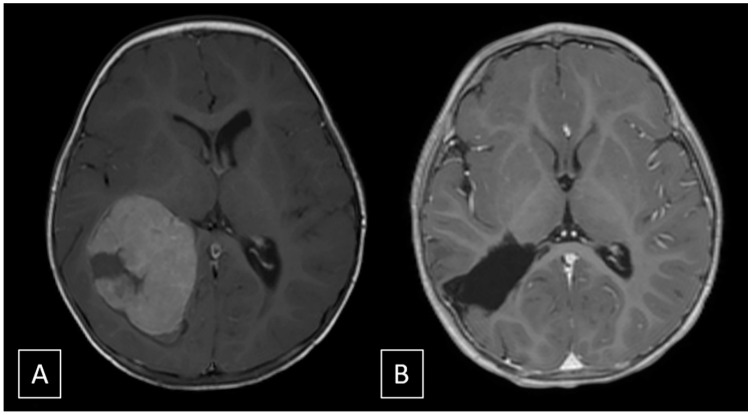
(**A**) T1 post-contrast image pre-operative shows a large enhancing intraventricular mass without invasion of the adjacent brain parenchyma. (**B**) T1 post-contrast image at 3-months follow-up shows absence of recurrence.

Immediately after the endovascular procedure, the patient underwent a surgical excision of the tumor. Opening of the right parietal craniotomy and dura revealed a herniation of the superior parietal lobule. The cortex was incised, leading to the spontaneous drainage of an intralesional haemorrhage and the identification of the neoplastic tissue, which was soft and scarcely bleeding. A gross total resection was easily achieved and an external drainage was left for 4 days postoperatively and then removed. Total blood loss was estimated to be approximately 200 cc, mainly during soft tissue opening. Histopathological analysis of the specimen was consistent with WHO grade III choroid plexus carcinoma (Immunophenotype S100: +; CAM 5-2: +; MNF116: +, MIB_1_ > 20%). The patient was discharged at 8 days with no neurological sequelae, and was planned for adjuvant chemotherapy according to CPT-SIOP 2009 recommendation (6 cycles alternating Cyclophosphamide and Carboplatin). Follow-up at 3 months revealed regular development of the child and absence of recurrent disease at MRI (Figure 3).

## 3. Discussion

The extent of resection is the only prognostic factor influencing the survival of patients affected by CPC [2,4,5]. The achievement of a total tumor removal is often undermined by their vascular nature, responsible for significant perioperative morbidity and mortality due to important blood loss in patients with a small circulating volume [4,6]. In this scenario, the accomplishment of a preoperative reduction of tumor vascularity is crucial. The use of neoadjuvant chemotherapy has proven to be valid for decreasing intraoperative blood loss; nevertheless, it requires time to be effective (from 2 to 5 cycles). In fact, it cannot be used in cases of patients with CPT and symptoms of intracranial hypertension, requiring urgent surgical treatment [7]. The first reports of preoperative embolization of CPT described very limited success, serving as a deterrent in the past decade [4,9]. Nevertheless, preoperative embolization has proven to be a safe procedure to achieve tumor devascularization, resulting in a significant reduction of intraoperative blood loss and improving outcomes [8]. However, the procedure is still considered hazardous both for the vascular tumor features and the population involved. CPT are supplied by the anterior choroidal artery and the posterior choroidal arteries, which also nourish eloquent areas. The cisternal portion of the anterior choroidal artery supplies the optic tract, the lateral part of the geniculate body, the posterior two-thirds of the posterior limb of the internal capsule, the globus pallidus, the origin of the optic radiations and the middle third of the cerebral peduncle, the ventrolateral thalamus, and the mesencephalon. The posterior choroidal arteries, divided into medial and lateral groups, supply the subthalamic nucleus, the midbrain, portions of the thalamus, including the medial geniculate nucleus, the lateral geniculate nucleus, the pulvinar of the thalamus, and, partially, the medial temporal structures [12,14]. Moreover, small children represent another important drawback due to their small and tortuous vessels, increasing the challenge in cannulating the feeding vessels and the risks of vascular dissection and thrombosis [4,9]. Furthermore, despite the hypervascular nature of these tumors, their vessels present very limited dilation. In addition, the younger age of the patients and their low-weight limits the amount of contrast medium and poses the problem of radiation dose. Despite modest success in older series [4,9], the literature discloses encouraging results (Supplementary Table S1). The rate of partial and complete embolization in the group of patients with CPT submitted to preoperative embolization was 76.5%, enabling a gross total resection in the vast majority of the patients with limited blood loss, regardless of the degree of embolization. Endovascular procedures were complicated by hemorrhage in 9.4% of the cases [8,15] and stroke in 3.1% [8]; mortality was 0%.

Embolization through the anterior choroidal artery represents a technical challenge due to its role in supplying critical motor areas, the relative lack of collaterals, and the unfavorable small caliber of vessels. Despite the abovementioned conditions depicting the anterior choroidal artery as a hostile vessel for endovascular navigation, embolization through it can be performed in safe conditions following some important key-points listed by Trivelatto et al.: (a) The anterior choroidal artery enters into the ventricle, passing the choroidal fissure, giving no more supply to the brain parenchyma. This point is identified as the plexal (or choroidal) point; (b) it is necessary to navigate the microcatheter beyond this landmark, positioning the microcatheter as close as possible to the tumor blush; and (c) it is recommended to inject the embolic agent carefully in order to avoid a potentially harmful reflux [12].

Few considerations have been made regarding other embolic agents used for this purpose, and previous authors have chosen metallic coils, gelatin sponges, cyanoacrylates and ethanol, as highlighted in Supplementary Table S1. Wang et al. reported a 28.6% occurrence of tumor bleeding related to the endovascular procedure, adopting tris-acryl gelatin microspheres. They explained these complications with two possible mechanisms: (a) rupture of some collateral vessels of the lesion, which received an increased blood flow after particles were injected from other residual feeders; and (b) rupture of residual tumor vessels due to an increased pression, originating after a continuous injection of the particles into a wedge catheter in a feeding artery. These considerations raise the concern about the use of particles in intra-axial lesion, whose feeders are more fragile than extra-axial tumor, and when the flow of the proximal feeding arteries is arrested due to a catheter wedged in small tumor feeders, with a caliber matching the size of the microcatheter, or when the tortuous tumor feeders are straightened by the microcatheter [15]. Trivelatto et al. described the first report of a successful Onyx™ embolization of choroid plexus papilloma through the anterior choroidal artery without complications. The choice of this embolic agent was attributed to the low precipitation rate, permitting a deep penetration into the small vessels of the tumor. Moreover, the injection of this material is more controlled, avoiding reflux toward the plexal point by virtue of its non-adhesive properties and good visualization under fluoroscopy. Nevertheless, it requires a prolonged injection time, as well as protracted fluoroscopy and radiation exposure, which could be not negligible in pediatric patients [12,16]. Furthermore, Onyx™ safety is not well established in children because of potential dimethyl sulfoxide toxicity [17]. NBCA is a fast-acting adhesive glue that has long been used in both the adult and pediatric populations [18]; it provides a rapid vascular bed penetration in a flow-directed fashion and, therefore, is especially suited for children.

**Figure 4:**
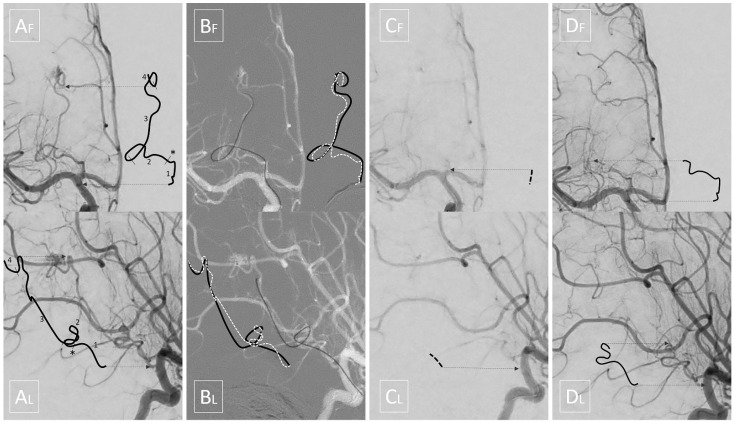
Upper quadrants display frontal views while lower quadrants display corresponding lateral views. Shifted lines are intended, as in Figure 1, to help the reader in localizing the intended segments. (**A**) Initial angiography: segment 1 is the cisternal portion of the anterior choroidal artery; the asterisk * corresponds to the plexal point that marks the entrance in the ventricle; segment 2 is the intraventricular segment which, in this case, is tortuous and ventrally dislocated by the tumor; segment 3 is the tumoral segment of the artery running along the surface of the tumor; segment 4 is the tumoral pedicle which penetrates the mass giving rise to the tumoral blush. (**B**) The black solid line depicts the arterial course stretched by the microcatheter, while the white dashed line depicts its anatomical course. (**C**) Panoramic angiography with the microcatheter inside the anterior choroidal artery; note that no flow is seen in the distal part of the cisternal segment of the anterior choroidal artery, and even the proximal part is barely visible. (**D**) Final angiography shows flow restored to the cisternal and intraventricular segments.

As mentioned above, we highlight a source of potential complication, which is insidious due to the specific pathology and populations affected. Indeed, it is otherwise infrequent for neurointerventional radiologists to navigate long segments of an eloquent artery that is only mildly enlarged by the underlying pathology (contrary to AVMs). Moreover, since contrast and dose sparing are required for pediatric interventions, it is also advisable to perform only strictly necessary control angiograms. Therefore, a potential absence of flow can be easily overlooked. Our case clearly demonstrates that the rectification of the artery, even using the smallest microcatheters and in the absence of spasm, may be sufficient to completely stop the flow along the whole course of the anterior choroidal artery, even proximally to the plexal point, where eloquent perforators are located (Figure 4). It is our opinion that the time from entrance to exit in the anterior choroidal artery should be monitored and regarded as a potential ‘occlusion time’ in this specific subset of patients. In fact, it may explain some of the complications reported in recent literature and it should be minimized, not only by the expertise, but also by the choice of fast embolic agents.

## 4. Conclusions

Previous medical literature remains inconclusive about the preferred embolic agent to be used in preoperative embolization of pediatric CPTs, with modern literature favoring liquid embolic agents. Given the rarity of the pathology, it is unlikely that definitive evidence will be available any time soon. Nevertheless, our case suggests that even simple navigation of the artery may cause temporary occlusion. Since prolonged occlusion of intracranial arteries may cause infarction, all efforts should be addressed to reduce navigation and embolization timings, thus favoring fast embolic agents, such as NBCA.

Although limited to a single observation, temporary arterial occlusion should be sought and considered a ‘red flag’ by neurointerventionalists, who should adapt their techniques accordingly.

## Data Availability

The data presented in this study are available on request from the corresponding author.

## Supplementary Materials

The following are available online at https://www.mdpi.com/article/10.3390/brainsci11050540/s1, Table S1: Review of patients with choroid plexus tumors submitted to embolization.
