# Supplementary material for: Preoperative Devascularization of Choroid Plexus Tumors: Specific Issues about Anatomy and Embolization Technique
Source: Brain Sci. 2021 Apr 25;11(5):540. doi: 10.3390/brainsci11050540 (PMC8146914; doi:10.3390/brainsci11050540)
Supplement: Supplementary file 1 [file brainsci-11-00540-s001.zip › brainsci-1164287-supplementary.pdf]

Table 1 Review of patients with choroid plexus tumors submitted to embolization

Author, year	N. pts EMB	Age	Sex	Localisation	Histology	Pre-op status	Cannulated artery	Material injected	Degree of embolisation	Complications	Time Emb to Surg	Degree of resection	Post-op status	Blood loss
Pencalet et al, 1998	7	NA	NA	Lateral ventricles	5 CPC, 2 CPP	NA	PChoA + steroptactic puncture	NA	Partial in 3 CPC and 1 CPP, failed in 3 cases	NA	NA	NA	NA	NA
Nagib et al, 2000	3	9 wks- 8 yrs	NA	Lateral ventricles	3 CPP	NA	NA	NA	Failed in all cases	None	NA	GTR	No deficit	NA
Otten et al, 2006	1	4 mths	M	Right atrium	CPP	Raised ICP, macrocephaly, full AF	AChoA	NBCA	Complete	None	1 day	GTR	right-gaze preference	200 ml
Takahashi et al, 2009	1	1 yr	F	Right lateral ventricle	Atypical CPP	Vomiting and lethargy. Previous surgery stopped because of excessive bleeding	Distal part of right AChoA	Spongel	Partial	None	1 day	GTR	No deficit	620 ml
Ditz et al, 2011	1	5 days	F	Right lateral ventricle	Atypical CPP	Macrocephaly	PChoA	Coils	Complete	None	1 day	GTR	No deficit	Minimal
Trivelato et al, 2012	1	10 mths	F	Right lateral ventricle	CPP	Left central facial palsy and left-sided paresis	AChoA	Onyx-18	Complete	None	2 days	GTR	The motor deficit improved	Minimal
Haliasos et al, 2013	15	15 days- 13 yrs	7 M; 6 F	Lateral ventricles (9 cases)	10 CPP, 5 CPC	NA	PChoA	Hystoacryl glue	5 complete, 8 partials, 2 failed	3/15: seizure, intratumoural haemorrhage, PCA stroke	6,5 days (everage)	GTR	NA	97.6%
Wang et al, 2013	4	1 mths	F	Left lateral ventricle	CPP	Macrocephaly	AChoA	NBCA	Complete	None	Same day	GTR	No deficit	180 ml
		5 mths	M		CPP	Macrocephaly	Medial PChoA	tris-acryl gelatin microsphe res	Complete	Hemorrhage	Same day	GTR	No deficit	500 ml
		8 mths	M		CPP	Macrocephaly	Lateral PChoA	tris-acryl gelatin microsphe res	Complete	None	Same day	GTR	No deficit	100 ml
		16 yrs	M		CPP	Seizures	AChoA, PChoA, MCA	tris-acryl gelatin microsphe res	Partial	Hemorrhage	1 day	STR	Left-sided weakness	1250 ml
Slater et al, 2016	1	1 yr	F	Right atrium	CPC	Macrocephaly, bulging AF, bradycardia, irritability	Anterior and Posterior	NBCA	Partial	None	2 days	GTR	NA	200 ml

							Choroidal arteries							
Our case	1	10 mths	F	Right atrium	CPC	Incidental diagnosis after TBI	AChoA	NBCA	Complete	None	Same day	GTR	No deficit	69.7% (200 ml)

Abbreviations AChoA: anterior choroidal artery; AF: anterior fontanel; CPC: choroid plexus carcinoma; CPP: choroid plexus papilloma; EMB: embolised; emb: embolization; GTR: gross total resection; yrs: years; MCA: medial cerebral artery; mths: months; NA: not available; NBCA: n-butyl 2-cyanoacrylate; pts: patients; PChoA: posterior choroidal artery; STR: subtototal resection; surg: surgery;
